# Preferences for breast cancer risk reduction among *BRCA1/BRCA2* mutation carriers: a discrete-choice experiment

**DOI:** 10.1007/s10549-017-4332-3

**Published:** 2017-06-17

**Authors:** Alexander Liede, Carol A. Mansfield, Kelly A. Metcalfe, Melanie A. Price, Kathleen Cuningham Foundation Consortium for Research into Familial Breast Cancer, Carrie Snyder, Henry T. Lynch, Sue Friedman, Justyna Amelio, Joshua Posner, Steven A. Narod, Geoffrey J. Lindeman, D. Gareth Evans

**Affiliations:** 1 Amgen Inc, South San Francisco, CA USA; 2 RTI Health Solutions, 200 Park Offices Drive, Research Triangle Park, NC 27709 USA; 3 Women’s College Hospital, University of Toronto, Toronto, Canada; 4 Centre for Medical Psychology and Evidence-based Decision-making (CeMPED), School of Psychology, The University of Sydney, Sydney, Australia; 5 Kathleen Cuningham Foundation Consortium for Research into Familial Breast Cancer (kConFab), Research Department, Peter MacCallum Cancer Centre, Melbourne, Australia; 6 The Sir Peter MacCallum Department of Oncology, University of Melbourne, Parkville, Australia; 7 Creighton University, Omaha, NE USA; 8 Facing Our Risk of Cancer Empowered (FORCE) Advocacy Organization, Tampa, FL USA; 9 Amgen Ltd, Uxbridge, UK; 10 The Royal Melbourne Hospital, Parkville, Australia; 11 Peter MacCallum Cancer Centre, Melbourne, Australia; 12 The Walter & Eliza Hall Institute of Medical Research, Parkville, Australia; 13 Manchester Centre for Genomic Medicine, University of Manchester, Manchester, UK

**Keywords:** *BRCA1*, *BRCA2*, Chemoprevention, Mastectomy, Genetic counseling, Risk perception

## Abstract

**Purpose:**

Unaffected women who carry *BRCA1* or *BRCA2* mutations face difficult choices about reducing their breast cancer risk. Understanding their treatment preferences could help us improve patient counseling and inform drug trials. The objective was to explore preferences for various risk-reducing options among women with germline *BRCA1/2* mutations using a discrete-choice experiment survey and to compare expressed preferences with actual behaviors.

**Methods:**

A discrete-choice experiment survey was designed wherein women choose between hypothetical treatments to reduce breast cancer risk. The hypothetical treatments were characterized by the extent of breast cancer risk reduction, treatment duration, impact on fertility, hormone levels, risk of uterine cancer, and ease and mode of administration. Data were analyzed using a random-parameters logit model. Women were also asked to express their preference between surgical and chemoprevention options and to report on their actual risk-reduction actions. Women aged 25–55 years with germline *BRCA1/2* mutations who were unaffected with breast or ovarian cancer were recruited through research registries at five clinics and a patient advocacy group.

**Results:**

Between January 2015 and March 2016, 622 women completed the survey. Breast cancer risk reduction was the most important consideration expressed, followed by maintaining fertility. Among the subset of women who wished to have children in future, the ability to maintain fertility was the most important factor, followed by the extent of risk reduction. Many more women said they would take a chemoprevention drug than had actually taken chemoprevention.

**Conclusions:**

Women with *BRCA1/2* mutations indicated strong preferences for breast cancer risk reduction and maintaining fertility. The expressed desire to have a safe chemoprevention drug available to them was not met by current chemoprevention options.

**Electronic supplementary material:**

The online version of this article (doi:10.1007/s10549-017-4332-3) contains supplementary material, which is available to authorized users.

## Introduction

Women who are *BRCA1* or *BRCA2* mutation carriers face high lifetime risks of breast and ovarian cancer. For *BRCA1* carriers, the risk of breast cancer ranges from 56% [1, 2] to 87% [3, 4], whereas for *BRCA2* carriers, the risk ranges from 33% [5] to 84% [6] by age 70. The risk of ovarian cancer ranges from 10% [1, 2, 5] to 60% [3–5] to age 70, depending on the gene and mutation location. Prospective observational data from the United Kingdom (UK) indicate that the risk of ovarian cancer is much higher among *BRCA1* carriers compared with *BRCA2* carriers (cumulative risk at age 70 years: 59 versus 17%) [2]. These high risks have been confirmed in prospective studies of unaffected *BRCA* carriers [7–10].

Risk-reducing bilateral mastectomy can decrease the risk of breast cancer by up to 95% [11], and risk-reducing bilateral salpingo-oophorectomy reduces the risk of ovarian cancer by approximately 80–90% [12–16]. In early studies, oophorectomy was associated with breast cancer risk reduction of approximately 50% [17], but recent findings call into question the benefit of oophorectomy for the prevention of premenopausal breast cancer in *BRCA1* mutation carriers [18, 19]. Tamoxifen has been associated with an approximately 50–70% reduction in the risk of contralateral breast cancer in *BRCA1/2* carriers [20, 21], but prospective studies of *BRCA1* carriers have not been done. Among unaffected women at high risk of breast cancer, a long-term follow-up study suggests an overall reduction of 37%, but there was no effect of tamoxifen on estrogen receptor–negative breast cancers [22]. Women can also rely on surveillance by undergoing frequent screening of the breasts [23–25] through digital or 3D mammograms or breast magnetic resonance imaging.

Once a mutation is identified, unaffected women face difficult decisions. Treatment decisions can increase cancer-related psychological distress [26, 27]. Surgical options carry physical and psychological risks, and oophorectomy has implications for fertility. Tamoxifen is associated with menopause-like symptoms and an increase in the risk of uterine cancer [22], and women are reluctant to take tamoxifen in part because they associate it with cancer treatment [47]. Furthermore, women of reproductive age are advised not to become pregnant while taking tamoxifen (although fertility is not adversely affected). Women in the Netherlands, the UK, and the United States (US) opt for mastectomy more often than do women from other countries, whereas tamoxifen uptake is consistently low in all countries [28, 29].

New studies evaluating receptor activated nuclear factor-κB ligand (RANKL) on breast cell proliferation have led to interest in a RANKL inhibitor as a possible chemoprevention alternative. RANKL-driven progesterone signaling has been shown to play a critical role in breast cancer tumorigenesis in *BRCA1* mutation carriers [18, 30–35].

Better information on women’s preferences for risk reduction and the tradeoffs they are willing to make between the benefits and risks of different options could help illuminate current treatment choice patterns and identify unmet needs, including acceptable chemoprevention options. In this study, we assessed the preferences for breast cancer risk-reduction treatments among women with germline *BRCA1* and/or *BRCA2* mutations. We also asked the women what preventive actions they had taken in the past and what actions they would have taken had other chemoprevention options been available to them. We compare the results of women’s preferences for hypothetical treatments with the actual choices they made to provide insights into treatment preferences.

## Methods

### Survey instrument

#### Women’s preferences

Following good research practices [36], we created a discrete-choice experiment (DCE) survey instrument that presented with a series of choices between two medicines with attributes that captured key benefits and risks associated with risk-reducing surgeries, tamoxifen, and a RANKL inhibitor with features similar to denosumab. The attributes were developed through consultation with clinical experts and a review of literature on breast cancer risk reduction. The final set of seven attributes and levels was as follows:

- Reduction in the risk of breast cancer (90, 75, 50, 40%)
- How long you take the medicine (1, 3, 5 years)
- Effect on ability to get pregnant (no effect, cannot get pregnant during treatment, can never get pregnant)
- Effect on female hormones (no effect, temporary menopause-like symptoms, early menopause)
- Risk of teeth and jaw problems (0, 1, 5%)
- Risk of getting uterine cancer (0, 1%)
- How you take the medicine (daily pill, injection at the doctor’s office every 3 months, injection at the doctor’s office every 6 months)

**Fig. 1:**
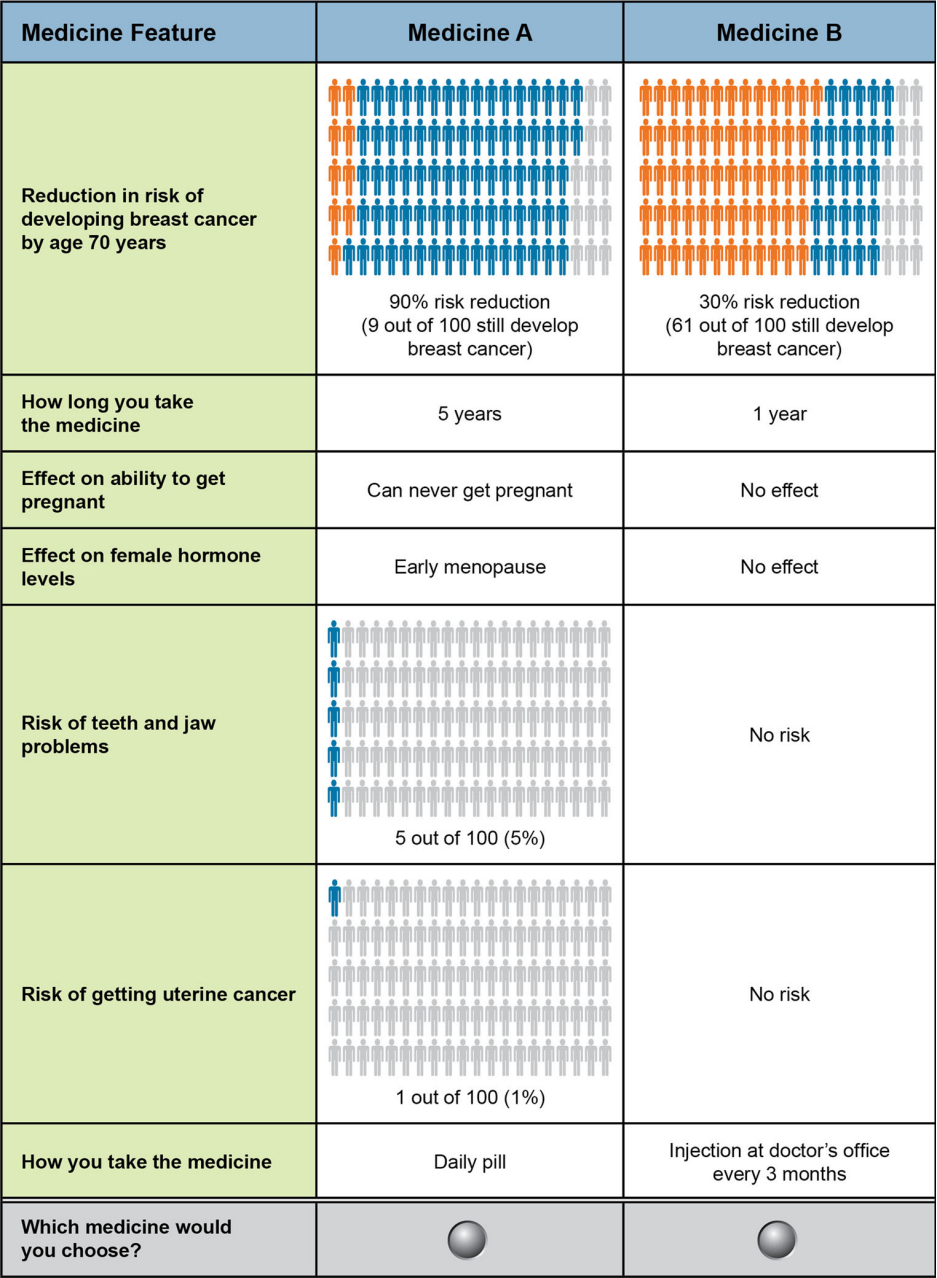
Sample discrete-choice experiment question

Each attribute was carefully explained prior to the medicine choice questions (see Table S1 [Supplement] for the attribute descriptions in the survey). Risk-related attributes were presented using risk grids with 100 figures [37]. The risk grids were explained, and respondents were asked questions testing their comprehension. Time since their genetic test disclosure ranged from several months to 23 years. When answering the DCE questions, women were asked to think back to when they first learned of their genetic status. Figure 1 provides a sample question.

The attribute levels were used to create a set of medicine choice questions based on an experimental design with known statistical properties. The design was created using a SAS implementation of a commonly used D-optimal algorithm to construct a fractional factorial experimental design [38–40] following best practice guidelines [41]. The full design contained 36 DCE questions offering a choice between two medicines. To limit respondent burden, nine blocks of four DCE questions were created, and each respondent was randomly assigned to see one of the nine blocks.

#### What women would have done

**Fig. 2:**
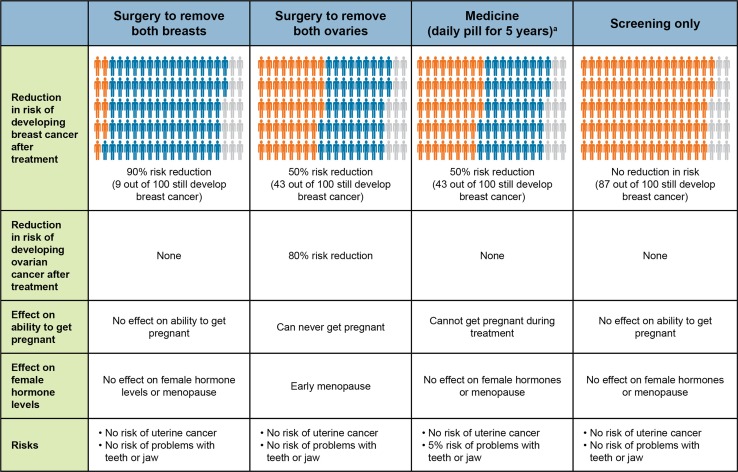
Treatment sequencing questions. ^a^Respondents were randomly assigned to Medicine 1 or Medicine 2. Medicine 1 is shown in the figure with features like a receptor activated nuclear factor-κB ligand (RANKL) inhibitor similar to denosumab. Medicine 2 had features like tamoxifen, i.e., the same attribute levels as Medicine 1 except a 40% reduction in the risk of breast cancer, a temporary effect on female hormones or menopause, a 1% risk of uterine cancer, and no risk of problems with teeth and jaw

Respondents were presented with three risk-reducing treatments (mastectomy, oophorectomy, and one of two hypothetical drugs) and screening (Fig. 2). The two hypothetical drugs had attributes related to a RANKL inhibitor or tamoxifen, and respondents were randomly assigned to one of the two drugs. In hypothetical choice questions, respondents were asked to think back to when their genetic mutation was first identified and indicate which treatments (including screening only) they would have selected.

#### Background questions and what women actually did

Demographic information (e.g., age, sex, education, employment status, race), risk perceptions, current level of distress (measured by the Modified Impact of Events Scale in Horowitz et al. [42] and Weiss and Marmar [43]), history with risk-reducing treatments, and relevant family medical history were also collected in the survey. The survey was pretested for comprehension and relevance in 14 semistructured interviews. Cultural adaptations to the survey were made for use in Canada, Australia, and the UK.

### Recruitment and eligibility

Women aged 25–55 years with an inherited mutation in the *BRCA1* and/or *BRCA2* gene who were unaffected by breast or ovarian cancer were eligible to participate in the survey. Respondents were recruited through a patient advocacy group, Facing Our Risk of Cancer Empowered (FORCE), and through the research registries at Creighton University (US), Women’s College Hospital (Canada), Royal Melbourne Hospital (Australia), Kathleen Cuningham Foundation Consortium for Research into Familial Breast Cancer (kConFab) at the Peter MacCallum Cancer Centre (Australia), and Manchester Centre for Genomic Medicine (UK). FORCE recruited respondents who provided a self-reported diagnosis of their *BRCA1/2* status through its website, newsletters, and social media. The clinical sites identified respondents who met the inclusion criteria and mailed them invitation letters with the URL of the online survey and a unique password. Institutional review boards at RTI International and all participating sites approved the study. All patients provided informed consent prior to their inclusion in the study.

### Statistical analysis

#### Women’s preferences

The DCE data were estimated using the random-parameters logit model to calculate the preference weights for all attribute levels using NLOGIT software, version 5.0 [44]. Random-parameters logit accounts for unobserved preference heterogeneity among respondents [45]. A main-effects model was estimated, which provided parameter estimates for each attribute level. The levels for the attributes’ “effect on ability to get pregnant,” “effect on female hormones,” and “how you take the medicine” were effects-coded [45], while the rest of the attributes were coded as linear, continuous variables.

Pretest interviews suggested that treatment duration may influence the preference for specific attributes, including ability to get pregnant, temporary menopause-like symptoms, and treatment-related risks. To test for interactions between duration and other attributes, an additional model was estimated, but the interaction terms were not statistically significant (*P* > 0.05), so the main-effects model was selected as the final model.

Planned subgroup analyses included women who planned to have children, women with first-degree relatives with breast or ovarian cancer, and women with higher education (college or higher). The subgroup analyses included interaction terms in the main-effects model. A Chi squared test was estimated to test for the joint significance of the interaction terms.

The preference weights from the random-parameters logit model were used to predict the share of the sample that would have selected hypothetical treatments in several scenarios. A hypothetical medicine with the characteristics of bilateral mastectomy or a bilateral oophorectomy was separately compared with two hypothetical medicines with characteristics like tamoxifen or an anti-RANKL monoclonal antibody.

#### What women would have done

The responses to questions that asked women whether they would have chosen mastectomy, oophorectomy, one of two medicines, or screening only were summarized by calculating the percentage of women who selected each option. A Chi squared test was used for comparison of percentages, and a *t* test was used for comparison of means.

## Results

Between January 2015 and March 2016, the clinical sites mailed 1163 letters to potentially eligible women, 383 women accessed the survey, and 338 women met the inclusion criteria. Of the 832 women from FORCE and the clinics who met the eligibility criteria, 622 respondents answered at least one of the DCE questions and were included in the analysis. Overall, the median age of the respondents was 41 years, and median age when women learned about their gene mutation was 37 years. Fifty-two percent of the women had a *BRCA1* mutation, 46% had a *BRCA2* mutation, and 1% had both *BRCA1* and *BRCA2* mutations (Table 1).

**Table 1:** Characteristics, actions taken, and actions planned

Characteristics of respondents with *BRCA1/2* mutations (*N* = 622)	Respondents or years
Site (country), No. (%)	
Creighton (US)	40 (6)
Manchester (UK)	118 (19)
Toronto (Canada)	31 (5)
kConFab (Australia)	79 (13)
Royal Melbourne Hospital (Australia)	45 (7)
FORCE (US)	309 (50)
Current age (years)	
Median	41.0
Mean (SD)	41.0 (8.2)
Marital status (*n* = 577), No. (%)	
Married/living as married/civil partnership	440 (76.3)
Single/never married	86 (14.9)
Divorced or separated	47 (8.1)
Widowed/surviving partner	1 (0.2)
Other	3 (0.5)
Employment status (*n* = 577), No. (%)	
Employed full time	329 (57.0)
Employed part time	106 (18.4)
Self-employed	56 (9.7)
Homemaker	55 (9.5)
Student	10 (1.7)
Retired	6 (1.0)
Disabled/unable to work	5 (0.9)
Unemployed	10 (1.7)
Gene mutation, No. (%)	
*BRCA1*	323 (51.9)
*BRCA2*	283 (45.5)
*BRCA1* and *BRCA2*	9 (1.4)
Don’t know or not sure	7 (1.1)
Years since *BRCA1/2* mutation was identified	
Median	4.0
Mean (SD)	4.8 (4.3)
Family history (*n* = 581), No. (%)	
Close relative with breast cancer before age 50 years	422 (72.6)
Close relative with ovarian cancer at any age	270 (46.5)
Two or more family members with breast cancer, either one relative with bilateral breast cancer or two or more relatives with breast cancer on the same side of the family	387 (66.6)
A male relative with breast cancer	43 (7.4)
Combination of breast, ovarian, and/or pancreatic cancer on the same side of the family	224 (38.6)
Three or more relatives with breast cancer at any age	269 (46.3)
None of the above	23 (4.0)

Actions taken to reduce breast or ovarian cancer risk by respondents with *BRCA1/2* mutations (*N* = 622)	Respondents, No. (%)
Mastectomy	306 (49.2)
Mastectomy without reconstruction	11 (1.8)
Mastectomy with reconstruction	295 (47.4)
Oophorectomy	325 (52.3)
Birth control pill	117 (18.8)
Prescription medication such as tamoxifen, raloxifene, or an aromatase inhibitor (anastrozole, exemestane)	34 (5.5)
Vitamin supplements or over the counter medicines (non-prescription)	119 (19.1)
Herbs or homeopathic medicines	33 (5.3)
Healthier lifestyle (improved my diet, exercised more, reduced stress, limited drinking alcohol)	253 (40.7)
Other risk reduction action	49 (7.9)
No risk reduction action	54 (8.7)
Annual breast screening (mammograms, MRI, or ultrasounds)	438 (70.4)
Started screening before age 40	415 (66.7)
Breast self-exams more frequently at home	377 (60.6)
Other early detection action	46 (7.4)
No early detection action	40 (6.4)

Actions planned or hypothetical to reduce risk of breast cancer risk by respondents with *BRCA1/2* mutations (*N* = 622)		Respondents, No. (%)
Likelihood to undergo mastectomy in the future (*n* = 310)^a^		
Very likely		132 (42.6)
Somewhat likely		65 (21.0)
Unlikely		38 (12.3)
Very unlikely		37 (11.9)
Don’t know or not sure		38 (12.3)
Likelihood to undergo oophorectomy in the future (*n* = 291)^b^		
Very likely		192 (66.0)
Somewhat likely		65 (22.3)
Unlikely		10 (3.4)
Very unlikely		10 (3.4)
Don’t know or not sure		14 (4.8)
Reasons for not taking tamoxifen, raloxifene, or an aromatase inhibitor (up to 3 selections) (*n* = 554)^c^		
My doctor did not recommend any of these medicines		287 (51.8)
I’m worried about the side effects of those medicines		217 (39.2)
I try not to take too many medicines		102 (18.4)
I have never heard of these medicine options		79 (14.3)
I cannot take those medicines because I want to get pregnant		77 (13.9)
I’m worried about the chance of blood clots or stroke		60 (10.8)
I know people who took those medicines to treat cancer, and I do not have cancer		53 (9.6)
I’m worried about the chance of developing uterine cancer		43 (7.8)
I don’t want to take a pill every day		35 (6.3)
Some other reason		111 (20.0)
What women would have done		
Ever selected (*n* = 598)	Mastectomy	441 (73.7)
	Oophorectomy	470 (78.6)
Ever selected (*n* = 289)^d^	Medicine 1^e^	90 (31.1)
Ever selected (*n* = 309)^d^	Medicine 2^e^	88 (28.5)

*FORCE* facing our risk of cancer empowered (advocacy organization), *kConFab* Kathleen Cuningham Foundation Consortium for Research into Familial Breast Cancer, *MRI* magnetic resonance imaging, *N* total sample size, *n* sample size for individual question if different from total, *SD* standard deviation, *UK* United Kingdom, *US* United States, *Mastectomy* risk-reducing bilateral mastectomy, *oophorectomy* risk-reducing bilateral salpingo-oophorectomy

^a^Among women who have not had a risk-reducing bilateral mastectomy

^b^Among women who have not had a risk-reducing salpingo-oophorectomy

^c^Among women who have never taken a prescription drug to reduce the risk of developing cancer

^d^Respondents were assigned to answer Medicine 1 or Medicine 2 choice questions from discrete-choice experiment

^e^Medicine 1 had attribute levels similar to a receptor activated nuclear factor-κB ligand (RANKL) inhibitor like denosumab, and Medicine 2 had attribute levels similar to tamoxifen

### Women’s preferences

**Fig. 3:**
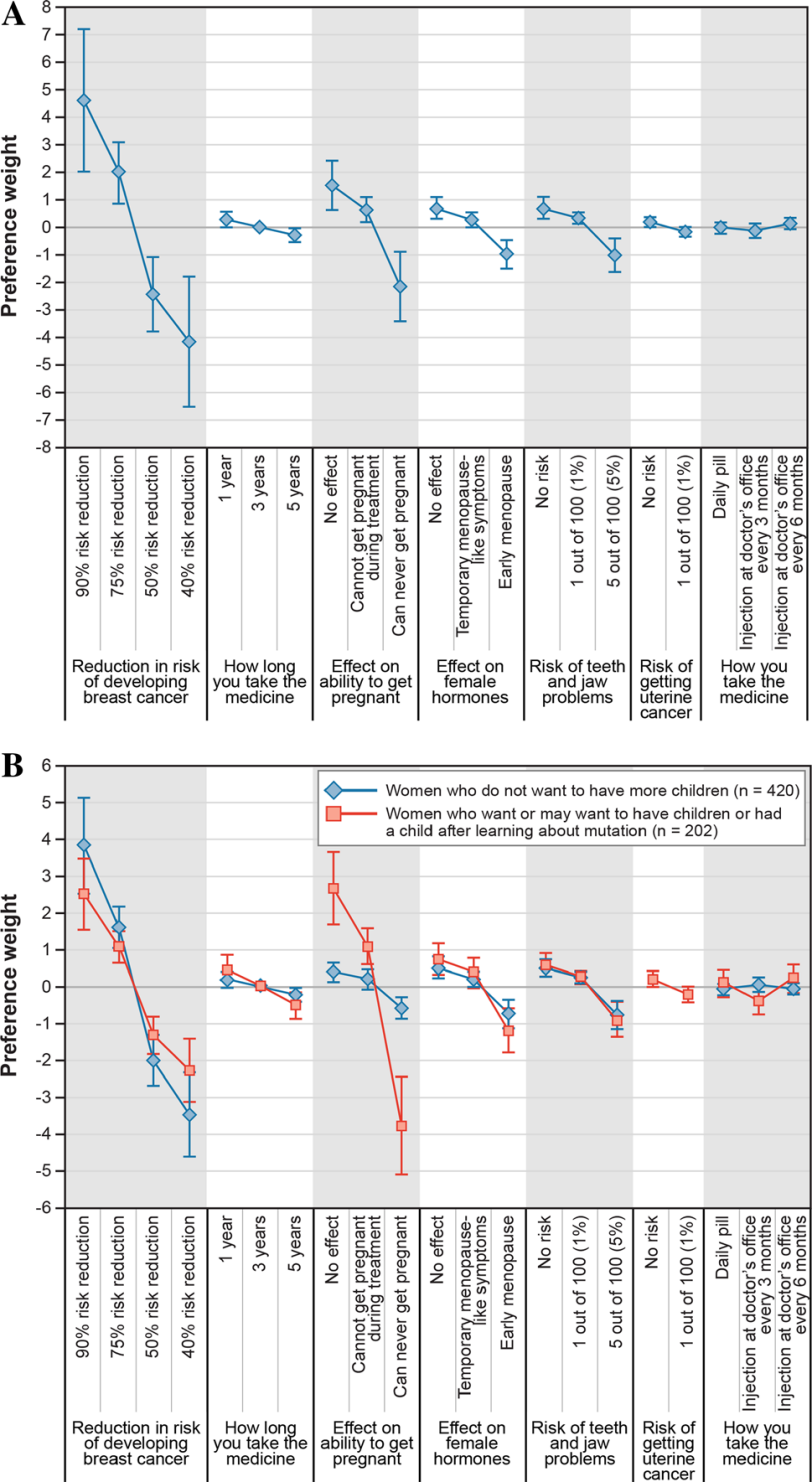
Normalized preference weights from random-parameters logit model. **a** Full sample. **b** Comparing women who wanted more children with women who did not. *Note* Included in the subgroup of women who wanted to have children were women who responded that they currently/in the future plan to have children as well as those who learned their *BRCA1/2* status before the birth of their first child. The *vertical bars* surrounding each mean preference weight denote the 95% confidence interval about the point estimate

Among the seven attributes characterizing the risk-reduction options in the DCE, women placed the most importance on breast cancer risk reduction, followed by the effect on ability to get pregnant. Respondents were indifferent between the three modes of administration, and a 1% risk of uterine cancer was less important than changes in the other attributes (Fig. 3a). The preference weights in Fig. 3 indicate the relative strength of preference for each attribute level, where larger positive numbers indicate greater preference and smaller negative numbers indicate less preference. The vertical distance between two levels of an attribute measures the relative strength of preference for the change in level.

The relative importance of the attributes depends on whether the woman wanted to have children at the time her genetic mutation status was identified, and the difference in preferences between the subgroups was statistically significant (*P* < 0.01). Women who wanted to have children placed the most importance on preserving their fertility (Fig. 3b). For women who did not want more children, an increase in the relative risk reduction from 40 to 90% was 7.6 times (95% CI 4.5–10.7) more important than a change from a treatment that left women with no ability to get pregnant to one that had no impact on the ability to get pregnant. For women who wanted to have children, the opposite was true—preserving fertility was 1.4 times (95% CI 1.1–1.6) more important than the largest increase in risk reduction. The preferences of the subgroup with a self-reported mutation status and those with a physician-confirmed status were not statistically significantly different, nor were the preferences of women with first-degree relatives who had breast or ovarian cancer or women with a college education (*P* > 0.05).

**Fig. 4:**
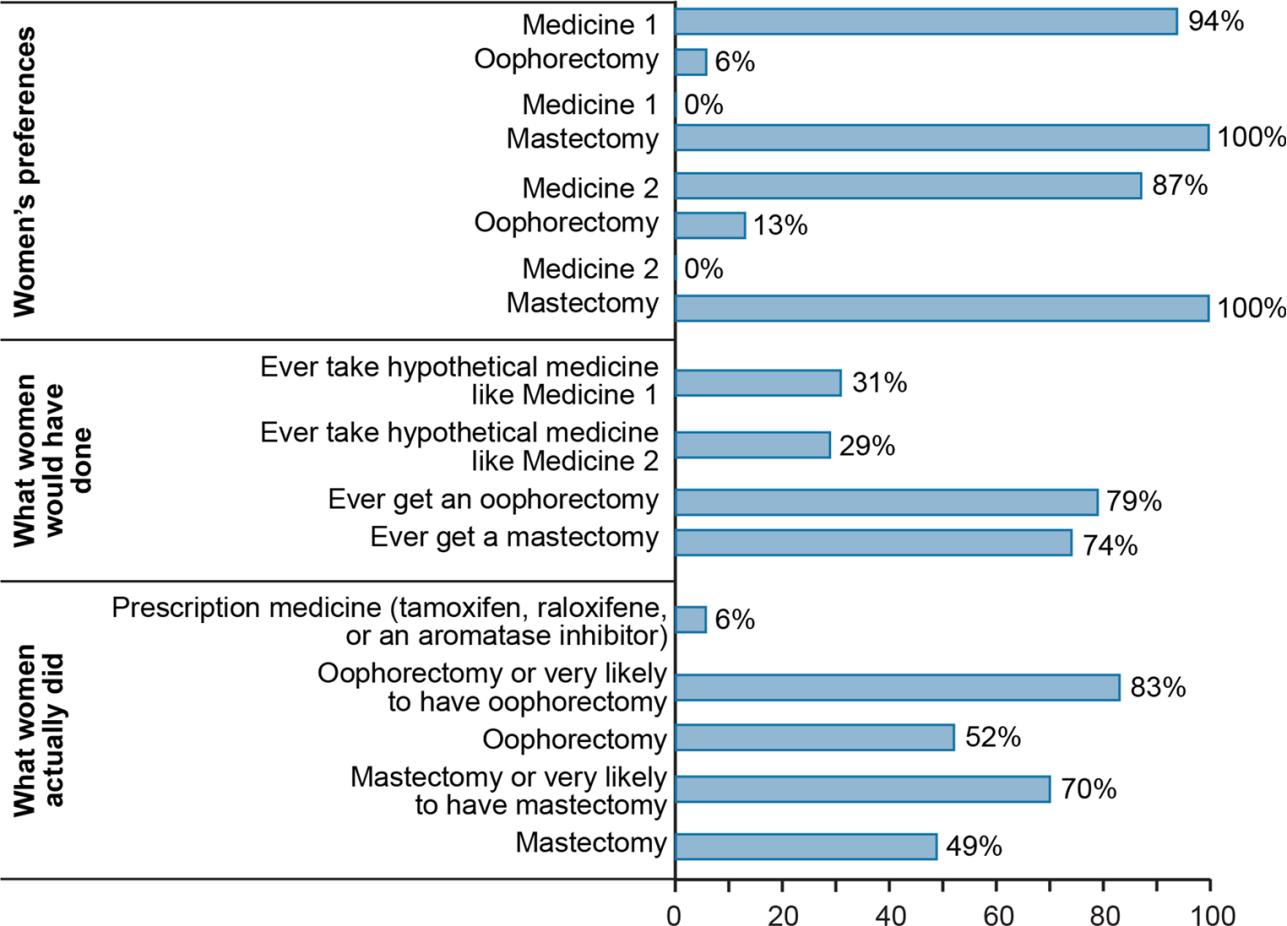
Summary of the reported preferences for risk-reducing bilateral mastectomy, oophorectomy, tamoxifen and RANKL inhibitor. *RANKL* receptor activated nuclear factor-κB ligand. *Note* For women’s preferences, estimates for share of sample preferring one option over another when pairs of options were evaluated were estimated from the full sample discrete-choice experiment results. The attributes of each option are defined as follows: Medicine 1 (similar to a RANKL inhibitor like denosumab): 40% risk reduction, cannot get pregnant during treatment, no effect on female hormone levels or menopause, 0% risk of uterine cancer, and 5% risk of teeth and jaw problems; bilateral oophorectomy: 50% reduction in risk of breast cancer, can never get pregnant, early menopause, and no risk of uterine cancer or teeth and jaw problems; bilateral mastectomy: 90% reduction in risk of breast cancer, no effect on ability to get pregnant, no effect on female hormone levels or menopause, and no risk of uterine cancer or teeth and jaw problems; Medicine 2 (similar to tamoxifen): 40% risk reduction, cannot get pregnant during treatment, temporary menopause-like symptoms, 1% risk of uterine cancer, and no risk of teeth and jaw problems. Duration of treatment and “how you take the medicine” were held fixed in these analyses

In Fig. 4, the top panel presents the percentages of the sample who would select medicines with features like mastectomy and oophorectomy compared with those who would select hypothetical Medicine 1 (a RANKL inhibitor similar to denosumab) and Medicine 2 (similar to tamoxifen) based on the DCE results for the full sample. A medicine with the risk-reducing properties of a mastectomy was preferred by the full sample over either of the chemoprevention options due to the importance of risk reduction overall.

### What women actually did

Forty-nine percent of the women had a risk-reducing mastectomy, 52% had an oophorectomy, 33% had both operations, and 32% had neither operation (Table 1). Women who had a *BRCA1* mutation were more likely to have had an oophorectomy than women who had a *BRCA2* mutation (57 versus 47%) (*P* = 0.02), whereas there was no significant difference in the percentage who had mastectomy by mutation (*P* > 0.05). Combining the women who had surgery with the women who had not had surgery but who said they would in the future brings the total to 70% for mastectomy and 83% for oophorectomy (Fig. 4, bottom panel).

Only 5.5% of the women in the study had taken a prescription medicine to reduce breast cancer risk (tamoxifen, raloxifene, or an aromatase inhibitor). When asked to select reasons for not having done so, the top reason was that their doctor had not recommended any of the medicines (52%), followed by concern about side effects (39%). Fourteen percent wished to get pregnant (Table 1).

In Fig. 4, the percentage of women who have had a surgery or who are likely to have a surgery is close to the percentage who said they would have surgery if they had to make the decision again. However, the percentage of respondents who said they would take a medicine like tamoxifen given the hypothetical choices was much greater than the percentage who had actually taken the drug.

### What women would have done

When women were asked to select all the measures they would have if they had their decisions to make again, 79% selected oophorectomy, 74% selected mastectomy, 31% selected Medicine 1 (similar to a RANKL inhibitor like denosumab), and 28% selected Medicine 2 (similar to tamoxifen) (Fig. 4).

## Discussion

Women with *BRCA1/2* mutations are willing to accept a risk of side effects in order to achieve a 90% reduction in breast cancer risk. The preferences expressed by the women in this survey were by and large consistent with the actions taken. Two-thirds of the women in the sample had undergone mastectomy, oophorectomy, or both, and among the remaining third of women who had not had surgery, at least half were planning on one or both surgeries in the future.

For women who wanted to have children, preserving fertility was the most important attribute out of the set of attributes studied. These women overwhelmingly preferred a hypothetical medicine that preserved fertility compared to an option that did not preserve fertility if the degree of protection was the same, even if there were more side effects. The DCE portion of the survey did not include ovarian cancer risk reduction as an attribute, and women reported that reducing the risk of both ovarian and breast cancer was the most important reason for having an oophorectomy. However, many women will delay oophorectomy because they wish to have more children. These results highlight a need for more risk-reducing options that preserve fertility, especially as genetic testing for *BRCA1/2* becomes more popular among young women.

The DCE included chemoprevention with attributes and levels based on a selective estrogen receptor modulator or a RANKL inhibitor. Recent findings point to the RANKL blockade, such as with denosumab, as a promising risk-reduction strategy that warrants further investigation for women who carry *BRCA1* or *BRCA2* mutations [34, 35, 46]. Denosumab, administered by injection, holds potential promise as a chemoprevention alternative in this population of otherwise healthy women who may undergo risk-reducing oophorectomy [47].

Low uptake and interest in tamoxifen/raloxifene for breast cancer risk reduction seen in our study is a consistent observation across studies and regions [28, 48]. Women have previously expressed concerns in addition to side effects that the medication would be a daily reminder of their risk, that tamoxifen was inextricably associated with cancer, and that tamoxifen chemoprevention evoked painful memories [48].

Women who are attempting to become pregnant should be counseled against taking tamoxifen or a RANKL inhibitor, although neither treatment should inhibit a woman’s ability to conceive should she wish to become pregnant after completing treatment. Effective contraception methods should be continued for approximately two months (tamoxifen) and five months (denosumab) after discontinuing these agents before attempting to conceive in order to avoid potential harmful effects to the fetus [49, 50].

Several limitations should be considered in the interpretation of our study results. The sample may not be representative of women with *BRCA1/2* generally. The DCE questions can only accommodate a limited number of attributes, so important attributes such as the risks associated with surgery and the level of ovarian cancer risk reduction were not included. Relative preferences for the attributes such as breast cancer risk reduction could be different if additional attributes had been included. Finally, past actions to reduce breast cancer risks were self-reported.

DCE studies provide a systematic method for ranking preferences for treatment options. This is the first study to implement the DCE methodology to provide a clear picture of the preferences among women with *BRCA1/2* mutations regarding the attributes of different options to reduce their risk of breast cancer. The preferences expressed in the survey were compared with actual actions and questions about what the women would have done if there had been other chemoprevention options available. Preferences for high levels of risk reduction and the ability to maintain fertility were dominant and reflected the actual choices women made.

Our survey indicated interest in chemoprevention, despite the low uptake of tamoxifen until now. Tamoxifen is not an acceptable option for chemoprevention for most women, in part due to its cancer-related stigma. A new chemoprevention option would be more acceptable to women who carry a *BRCA1/2* mutation. Future research is warranted to determine the efficacy and safety of RANKL inhibitors in this population.

## Electronic supplementary material

Below is the link to the electronic supplementary material.

Supplementary material 1 (DOCX 13 kb)
