# Supplementary material for: Preferences for breast cancer risk reduction among BRCA1/BRCA2 mutation carriers: a discrete-choice experiment
Source: Breast Cancer Res Treat. 2017 Jun 17;165(2):433–44. doi: 10.1007/s10549-017-4332-3 (PMC5543193; doi:10.1007/s10549-017-4332-3)
Supplement: Supplementary file 1 — Supplementary material 1 (DOCX 13 kb) [file 10549_2017_4332_MOESM1_ESM.docx]

Table S1. Attributes and Levels in the Treatment Choice Questions

| Attribute | Description From Survey Instrument | Level |
| --- | --- | --- |
| Reduction in risk of developing breast cancer by age 70 | The risk of breast cancer each woman faces depends on the type of mutation and other personal and family characteristics. For women with *BRCA1* and/or *BRCA2* mutations, the risk for developing breast cancer by the time they are 70 years old can be between 45% and 87%. | 90% risk reduction |
|  |  | 75% risk reduction |
|  |  | 50% risk reduction |
|  |  | 40% risk reduction |
| How long you take the medicine | We will show you medicines that you can take for different lengths of time, between 1 and 5 years. While you are taking the medicine, you would need to be examined by your doctor and have a mammogram or MRI every 6 months (2 times each year). After the treatment period has ended, you would be able to choose other treatment options. | 1 year |
|  |  | 3 years |
|  |  | 5 years |
| Effect on ability to get pregnant | Some treatments that reduce the risk of breast cancer can have an effect on whether you are able to get pregnant. We will ask you to consider hypothetical medicines that could have 1 of 3 effects on whether or not you can get pregnant. | No effect |
|  |  | Cannot get pregnant during treatment |
|  |  | Can never get pregnant |
| Effect on female hormone levels | Some treatments that reduce the risk of breast cancer can change the level of female hormones, estrogen and progesterone, in your body. The symptoms of these hormonal changes are similar to the symptoms of menopause and are different for every woman, but they can include the following:  Hot flashes (or hot flushes)  Decreased libido (sex drive) and other sexual side effects such as vaginal dryness  Sleep disturbances  Changes in mood  Changes in weight  Changes in memory | No effect |
|  |  | Temporary menopause-like symptoms |
|  |  | Early menopause |
| Risk of teeth and jaw problems | Some medicines can cause problems with your teeth and/or jaw bone, even if you have false teeth. These potential problems are caused by a condition called osteonecrosis of the jaw (ONJ). ONJ occurs when you have a wound in the gums that does not heal properly and exposes the jawbone inside the mouth, usually after a tooth is removed. The exposed area of the bone is usually painful. People with poor dental health have a greater risk of having problems with their teeth and/or jaw.  Other symptoms of ONJ include numbness, swelling, and a feeling of heaviness of the jaw. Jawbones can heal on their own with antibiotic rinses, but healing is not guaranteed.  Serious problems related to ONJ can be avoided. People are encouraged to maintain good dental health and to avoid major dental procedures such as having a tooth removed when taking the medicine. The risk that you would develop ONJ ends when you stop taking the medicine. | No risk |
|  |  | 1 out of 100 (1%) |
|  |  | 5 out of 100 (5%) |
| Risk of getting uterine cancer | Cancer in the uterus (also called cancer of the womb) is very uncommon for all women—fewer than 1 out of every 1,000 women (less than one-tenth of 1%) will get this type of cancer. Women who have uterine cancer usually find the cancer during a doctor’s exam, because of vaginal bleeding that is not related to a period, or because of pain during sex. Most of the time, uterine cancer can be successfully treated by having a surgery to remove the uterus, by chemotherapy, or by radiation.  Some medicines that reduce the risk of developing breast cancer also may cause a small increase in the risk of developing cancer in the uterus or endometrium (the lining of the womb). We will ask you to think about medicines that would increase the risk of uterine cancer to be 1%. The increased risk that you would develop uterine cancer ends when you stop taking the medicine. | No risk |
| How you take the medicine | Medicines that reduce the risk of breast cancer can be taken in different ways. In this part of the survey, we will describe medicines that are taken 3 different ways and for different lengths of time.  **Daily pill:** You have to take 1 pill by mouth every day.  **Injection at doctor’s office every 3 months:** Every 3 months (4 times each year), you need to go to a doctor’s office to get an injection into the skin on your upper arm. The injection will feel like a flu vaccine.  **Injection at doctor’s office every 6 months:** Every 6 months (2 times each year), you need to go to a doctor’s office to get an injection. The injection will be similar to the one described above. | Daily pill |
|  |  | Injection at doctor’s office every 3 months |
|  |  | Injection at doctor’s office every 6 months |
